# Thermal Balance Analysis of a Micro-Thermoelectric Gas Sensor Using Catalytic Combustion of Hydrogen

**DOI:** 10.3390/s140101822

**Published:** 2014-01-21

**Authors:** Daisuke Nagai, Takafumi Akamatsu, Toshio Itoh, Noriya Izu, Woosuck Shin

**Affiliations:** AIST, 2266-98 Anagahora, Shimo-Shidami, Moriyama-ku, Nagoya 463-8560, Japan; E-Mails: d-nagai@aist.go.jp (D.N.); t-akamatsu@aist.go.jp (T.A.); itoh-toshio@aist.go.jp (T.I.); n-izu@aist.go.jp (N.I.)

**Keywords:** thermoelectric gas sensor, combustion catalyst, thermal balance

## Abstract

A thermoelectric gas sensor (TGS) with a combustion catalyst is a calorimetric sensor that changes the small heat of catalytic combustion into a signal voltage. We analyzed the thermal balance of a TGS to quantitatively estimate the sensor parameters. The voltage signal of a TGS was simulated, and the heat balance was calculated at two sections across the thermoelectric film of a TGS. The thermal resistances in the two sections were estimated from the thermal time constants of the experimental signal curves of the TGS. The catalytic combustion heat *Q_catalyst_* required for 1 mV of *ΔV_gas_* was calculated to be 46.1 μW. Using these parameters, we find from simulations for the device performance that the expected *Q_catalyst_* for 200 and 1,000 ppm H_2_ was 3.69 μW and 11.7 μW, respectively.

## 1. Introduction

Inflammable gases such as CO, CH_4_, and H_2_, which can amount to several hundred parts per million in human breath [1,2], can be used for medical examination and detected by the micro-calorimetric device of a thermoelectric gas sensor (TGS) with a combustion catalyst. The TGS can be a useful platform device, because it is possible to modify the catalyst of the TGS for the target gas. We have reported that a TGS with a Pt-loaded alumina (40 wt%Pt/alumina) catalyst can detect H_2_ over a wide concentration range from as low as 0.5 ppm up to 5 vol.% H_2_ in air [3]. In addition, this device showed a good linearity between the H_2_ concentration in air and the sensing signal at the catalyst temperature of 100 °C. We have succeeded in measuring H_2_ in the human breath at the parts per million level [4]. Additional improvements in the TGS device are required for the detection of CO and CH_4_, such as much higher catalyst temperature and precise temperature control, because CO and CH_4_ are less inflammable as compared to H_2_. The thermal design of a TGS should be improved for efficient transport of the catalytic combustion heat of CO and CH_4_. In order to produce an effective design, the heat balance of the sensor during operation needs to be estimated. Moreover, the heat balance can predict the inflammable gas combustion energy of the catalyst, as the heat as a function of the sensor output is also important for the development of the catalyst. However, the heat of combustion of the small amount catalyst which is used for gas sensors is difficult to estimate since it is difficult to measure the temperature of small parts of devices like sensors.

In this paper, a calculation for the heat balance of both ends of the thermoelectric (TE) film of a sensor device is presented, and the sensor output is simulated. Calculations for the rate of catalytic combustion converted to a voltage signal using a heat balance calculation are compared with the experimental results that estimate the rate of catalytic combustion of a TE hydrogen sensor.

## 2. TGS Device Preparation

**Figure 1.**
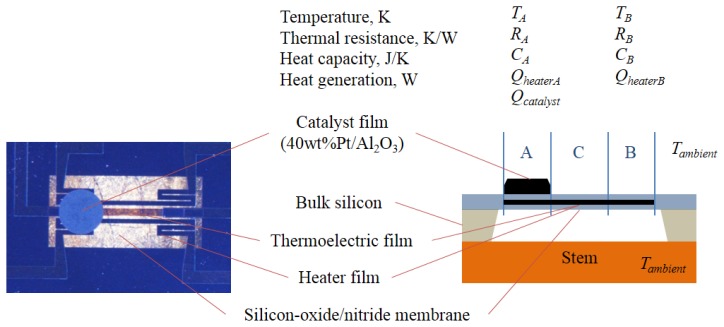
Optical image of the TGS (on the stem). Cross-sectional view of the TGS on the stem. Two sections of the heater meander line on the membrane are called sections A and B, and the center section of straight heater line is called section C.

Figure 1 shows a photograph of the micro-TGS with the ceramic combustion catalyst used in this study. A double-sided polished Si substrate with a thickness of 0.35 mm was used. A silicon-germanium (SiGe) thin film was deposited by DC magnetron sputtering and patterned into the TE material by RIE etching. Micro-heater and electrode lines were fabricated using a lift-off technique involving platinum. To fabricate a membrane structure, the bottom of the substrate was etched using an aqueous KOH solution. The detailed process has been previously reported [5]. A metal colloid solution was stirred constantly, followed by the addition of Al_2_O_3_ powder to the solution to create a catalyst powder of 40 wt% metal content. Then, distilled water was added to the solution, and the solution was agitated at 70 °C until the water evaporated. The solid residue obtained was dried at 90 °C for 30 min and then baked in air at 300 °C for 2 h to obtain the catalyst powder. A ceramic paste of the catalyst was prepared by mixing terpineol, ethyl cellulose, and distilled water at a weight ratio of 9:1:5. A drop of the ceramic paste was placed on the thin membrane of the micro-TGS using an air dispenser. The size of the drop on the membrane could be controlled to 0.6 mm in diameter by changing the dispensing time and air pressure. After paste deposition, the device was baked in air at 300 °C for 2 h.

Table 1 lists the material constants and boundary conditions for the TGS used in this study. The heat capacity of the dispensed 40 wt%Pt/Al_2_O_3_ catalyst, *C_catalyst_*, was 4.34 μJ/K, as estimated from the specific heat capacity, density and volume. The catalytic specific heat capacity was estimated from the catalytic constituent and material constants for Pt and Al_2_O_3_. The dimensions of the dispensed catalyst, diameter, thickness, and volume were measured using a P16+ Profiler manufactured by KLA-Tencor. The heat capacities of the membrane, *C_membrane_*, in sections A and B were assumed to be the same as *C_catalyst_* since the size of the membrane and the size of the catalyst are similar. *C_A_* was computed as the sum of *C_catalyst_* and *C_membrane_* and is equal to 8.68 μJ/K. *C_B_* was simply equal to *C_membrane_*, which is 4.34 μJ/K. *α* was 0.2 mV/K in this study, as obtained from [3].

The gas response of the sensor was investigated using a flow chamber. After placing a sensor in the flow chamber, air and the hydrogen were allowed to alternately flow into the chamber. The operating temperature was adjusted by heater control and the cold-side junction was monitored using an IR camera (Nikon, LAIRD-270A). The voltage signal from the sensor was monitored using a digital multimeter (KeithleyK2700).

## 3. Design and Detection Principle for the Calorimetric Device of a TGS

### 3.1. Thermal Balance in a TGS

A TGS consists of a thermal sensor that detects the temperature difference. The heat balance for a thermal sensor is briefly denoted by the following equation [8–10]:

(1) $$P=\left[T\left(t\right)-T_{\text{ambient}}\right]\times R^{-1}$$

The temperature difference *T*(*t*)−*T_ambient_* depends linearly on the thermal resistance *R* and heating power input *P*. A sensor with a low heating power output and big thermal resistance leads to a large temperature difference. In the TGS, the hot and cold junctions of a TE film are on a membrane, which can reduce unwanted noise caused by air flow, for example. Therefore, the heat balances in two junctions of a TE film become important and differ from each other in Equation (1). A TGS emits a signal *V_S_* according to the temperature difference *ΔT* between both ends of the TE film; *ΔT* is determined by two energy balances of the TE film at both ends.

Figure 1 shows an optical image of the TGS device [3]. Two sections of the heater meander line on the membrane are called sections A and B, and the center section of the straight heater line is called section C. The two different temperatures at the heater meander sections A and B in the membrane are temperatures *T_A_* and *T_B_*, respectively, of the ends of the TE film in the lumped parameter system. *V_S_* of the TGS can be expressed as follows from the Seebeck effect:

(2) $$V_{S}=\alpha \times \Delta T_{AB}=\alpha \times \left(T_{A}-T_{B}\right)$$

where*α* is the Seebeck coefficient of the TE film on the device. The influence of the temperature dependency of the Seebeck coefficient *α*(*T*) can be neglected since the temperature shift is small in combusting low concentration gas. The heat balance of the hot section A and cold section B can then be expressed as follows. Two thermal energy balance equations are written as:

(3) $$C_{A}\frac{dT_{A}\left(t\right)}{dt}=Q_{\text{heater}}+Q_{\text{catalyst}}-\frac{T_{A}\left(t\right)-T_{\text{ambient}}}{R_{A}}$$

(4) $$C_{B}\frac{dT_{B}\left(t\right)}{dt}=Q_{\text{heater}}-\frac{T_{B}\left(t\right)-T_{\text{ambient}}}{R_{B}}$$

where *C_A_* and *C_B_* are the heat capacities of sections A and B, respectively; *t* is the time; *Q_heater_* is the heat generation of the heater; *T_ambient_* is the ambient temperature; *Q_catalyst_* is the heat of the catalyst by inflammable gas combustion; and *R_A_* and *R_B_* are thermal resistances of conduction and convection to the ambient temperature of sections A and B, respectively. *R_A_* and *R_B_*—are constants since the gas flow velocity is constant.

### 3.2. Voltage Signal ΔV_gas_ for Inflammable Gas

The voltage signal Δ*V_S_* for combustion gas is the voltage difference between the saturated voltage of the TGS in inflammable gas *V_gas_* and that in air *V*_air_:

(5) $$\Delta V_{S}=V_{\text{gas}}-V_{\text{air}}$$

When the TGS signal is saturated, the TGS is considered to be in a thermally steady state. Thus, the left-hand sides of Equations (3) and (4) can be set to zero at steady state (*dT*/*dt* = 0), and be rewritten as follows:

(6) $$T_{A}=R_{A}\times \left(Q_{\text{heater}}+Q_{\text{catalyst}}\right)+T_{\text{ambient}}$$

(7) $$T_{B}=R_{B}\times Q_{\text{heater}}+T_{\text{ambient}}$$

To determine *V_gas_*, Equations (6) and (7) are substituted into Equation (2):

(8) $$V_{\text{gas}}=\alpha \times \left[R_{A}\times \left(Q_{\text{catalyst}}+Q_{\text{heater}}\right)-R_{B}\times Q_{\text{heater}}\right]$$

In order to solve for *V_air_* as in Equation (8), Equations (6) and (7) are substituted into Equation (2), where *Q_catalyst_* in Equation (6) is set to zero in air:

(9) $$V_{\text{air}}=\alpha \times Q_{\text{heater}}\times \left(R_{A}-R_{B}\right)$$

In order to calculate Δ*V_gas_*, Equations (8) and (9) are substituted into Equation (5):

(10) $$\Delta V_{\text{gas}}=\alpha \times Q_{\text{catalyst}}\times R_{A}$$

The parameters that determine Δ*V_gas_* for the TGS are *α*, *Q_catalyst_*, and *R_A_*.

### 3.3. Voltage Signal V_S_(t)

If we consider that Equations (3) and (4) are a first-order system response, these equations can be changed into the following by Laplace analysis [8]:

(11) $$T_{A}\left(t\right)-T_{\text{ambient}}=\left(Q_{\text{heater}}+Q_{\text{catalyst}}\right)\times R_{A}\times \left[1-\exp\left(\frac{-t}{R_{A}\cdot C_{A}}\right)\right]$$

(12) $$T_{B}\left(t\right)-T_{\text{ambient}}=Q_{\text{heater}}\times R_{B}\times \left[1-\exp\left(\frac{-t}{R_{B}\cdot C_{B}}\right)\right]$$

Then, the thermal time constants *τ_A_* and *τ_B_* are expressed as follows:

(13) $$\tau_{A}=R_{A}\times C_{A}$$

(14) $$\tau_{B}=R_{B}\times C_{B}$$

If Equations (11) and (12) are substituted into Equation (2)*V_S_* (*t*) is calculated as

(15) $$V_{S}\left(t\right)=\alpha \times \left\{\left(Q_{\text{catalyst}}+Q_{\text{heater}}\right)\times R_{A}\times \left[1-\exp\left(\frac{-t}{R_{A}\times C_{A}}\right)\right]-Q_{\text{heater}}\times R_{B}\times \left[1-\exp\left(\frac{-t}{R_{B}\times C_{B}}\right)\right]\right\}$$

The time constant of the voltage signal *τ_S_* in Equation (15) is determined by the correlation of sections A and B. Furthermore, *τ_S_* depends only on section A, indicating that *τ_S_* = *τ_A_* when the sensor is thermally stabilized by heating, and the combustion gas is introduced.

## 4. Experimental Verification and Discussions

### 4.1. Voltage Signal Response V_S_(t) of the TGS for Hydrogen Gas

The *Q*_catalyst_ is proportional to the hydrogen concentration of the air flow [3]. It is assumed that the catalyst combustion energy *Q*_catalyst_ is the constant in Equations (3) and (4). In TGS, the catalyst combustion energy is proportional to the gas concentration such as the H_2_. It takes time that the gas concentration in the chamber reaches the gas concentration of the flow to the chamber when the certain gas concentration flows to the chamber. So, the catalyst combustion heat is not the ideal constant parameter since the gas concentration in the chamber is not constant.

**Figure 2.**
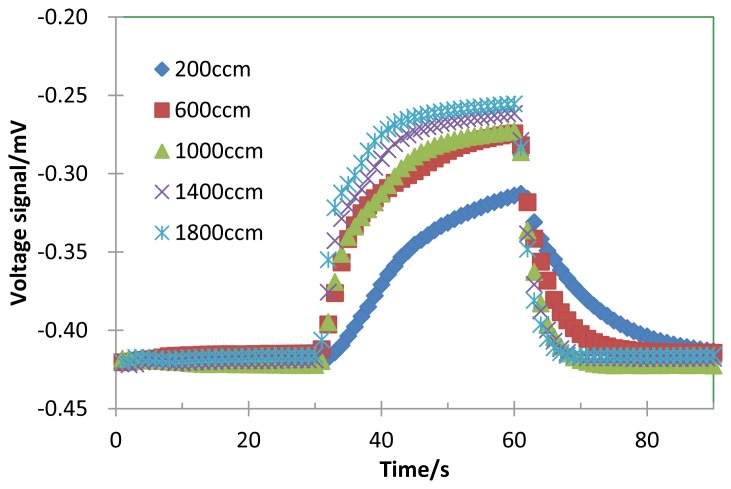
Experimental response curve of the TGS for 200 ppm H_2_ and the flow rate from 200 ccm to 1,800 ccm. The thermal time constant, measured as the elapsed time from 60 s to decreasing at 63.2% of the Δ*V_gas_*. The catalyst of the TGS device was heated up to 120 °C by its micro-heater. H_2_ flow was from 30 to 60 s.

Figure 2 shows the voltage signal response curve of the TGS for 200 ppm H_2_. The catalyst of the TGS device was heated up to 120 °C by its micro-heater. First, air flowed up to 30 s, then H_2_ flowed from 30 to 60 s, and then air flowed again, where the flow rate was changed from 200 to 1,800 ccm. The time constant is small by the gas mass flow because the time until the hydrogen concentration in the chamber reaches to 200 ppm is short.

### 4.2. Combustion Reaction Limited V_S_(t) of the TGS at High Gas Flow End

**Figure 3.**
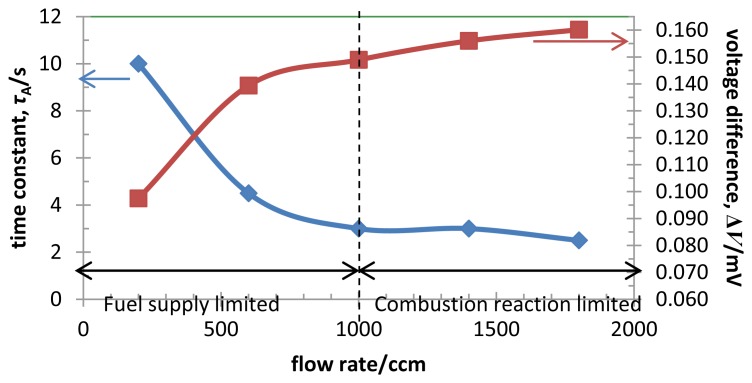
The flow rate dependence of the time constant, *τ*, and the voltage difference, Δ*V*_gas_, at the TGS for 200 ppm H_2_ in air and the flow rate from 200 ccm to 1,800 ccm.

Figure 3 shows the flow rate dependence of the thermal time constant, *τ*_A_, and the voltage difference, Δ*V*_gas_, of the TGS for 200 ppm H_2_ combustion under various gas flow rate from 200 to 1,800 ccm. *τ_A_* measured at the elapsed time from 60 s to decreasing at 63.2% of the saturated value of Δ*V_gas_*. For the gas flow rate below 1,000ccm, *τ*_A_ decreased from 10 to 3 s with the flow rate, and became constant over 1,000 ccm. *τ*_A_ will be independent of the flow rate in the reaction limited state.

In Figures 2 and 3, *ΔV*_gas_ increased with the flow rate. If the operating temperature is high enough to activate the combustion, the reaction would be diffusion-limited, being controlled by the diffusion of the hydrogen gas to the catalyst surface.

### 4.3. Estimation of Thermal Time Constants

**Figure 4.**
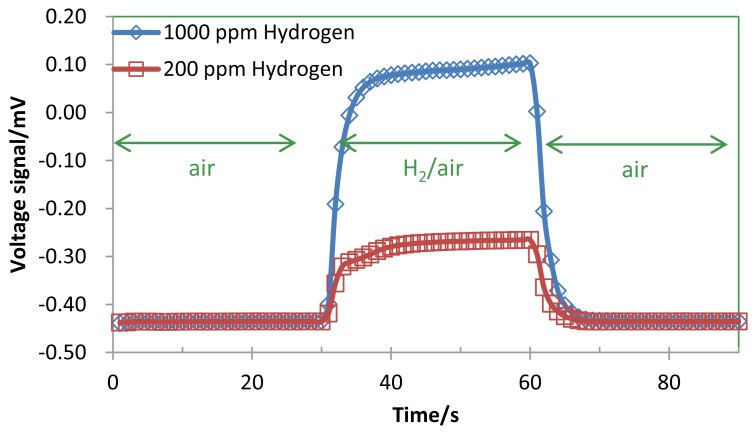
Experimental response curve of the TGS in the gas at a flow rate of 1,800 ccm of 200 ppm and 1,000 ppm H_2_ in air. The catalyst of the TGS device was heated up to 120 °C by its micro-heater. H_2_ flow was from 30 to 60 s. The thermal time constant *τ*_A_, measured as the elapsed time from 60 s to decreasing at 63.2% of the Δ*V_gas_* is 2.0 s.

Figure 4 shows the response curves and Δ*V_gas_* of the TGS for 200 ppm and 1,000 ppm H_2_ in air. Table 2 lists the estimated parameters from the experimental response curves in Figure 4. The catalyst of the TGS device was heated up to 120 °C by its micro-heater with a heater power of 50.0 mW.

Assuming that the thickness of Pt heater pattern on the TGS is constant, *Q_heaterA_* and *Q_heaterB_* were designed to be the same and were estimated to be 10.0 mW from the dimensions of the Pt heater pattern measured by the optical microscope image and Joule's laws. As shown in Figure 4, the thermal time constants of two signal curves were the same and equal to 8 s. The gas flow was introduced at steady state after the micro-heater had stabilized. We can then regard the time constant of 2.0 s as *τ_A_*, as described in Equation (15). From *C_A_* in Table 1 and the thermal time constant of section A, *τ_A_* = *R_A_* × *C_A_*, *R_A_* was estimated to be 230.4 K/mW. Using this value for *τ_A_* and *V_air_* = −0.435 mV in Figure 4, *R_B_* was estimated as 230.6 K/mW, and *τ_B_* was calculated as 1 s. *Q_catalyst_* can be calculated from Equation (10) as follows:

(16) $$Q_{\text{catalyst}}=\frac{\Delta V_{\text{gas}}}{\alpha \times R_{A}}=0.0217\;[W/V]\times \Delta V_{\text{gas}}$$

*Q_catalyst_* required for 1 mV of Δ*V_gas_* was calculated to be 21.7 μW. The reciprocal of the coefficient of 46.1 in Equation (16) is a parameter representing voltage per unit catalytic combustion heat [V/W] that represents the efficiency of the TGS device. Δ*V_gas_* of the TGS for 200 and 1,000 ppm H_2_ was 0.170 and 0.537 mV, respectively, and the expected *Q_catalyst_* using Equation (16) for 200 and 1,000 ppm H_2_ was estimated as 3.69 μW and 11.7 μW, respectively. An enthalpy of hydrogen combustion is 286 kJ/mol. The molar quantity of hydrogen combustion of TGS is 3.35 × 10^−6^ mol/s since the heat generation of 1,000 ppm hydrogen combustion is 11.7 μW.

### 4.4. Simulated Voltage Signal Response V_S_(t)

**Figure 5.**
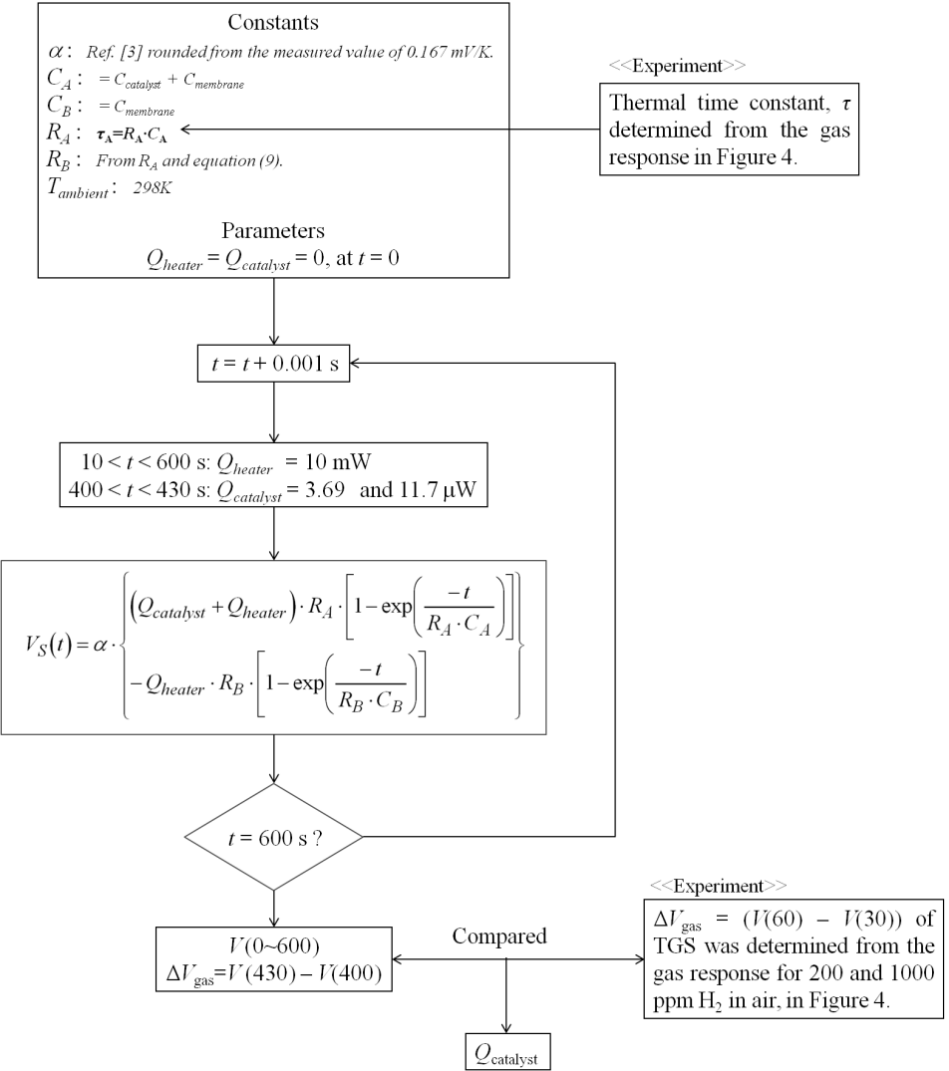
Flow chart for the simulation for calculating the voltage signal *V_S_*(*t*) in Equation (15) of TGS compared to the experimental response Δ*V*_gas_ of TGS.

**Figure 6.**
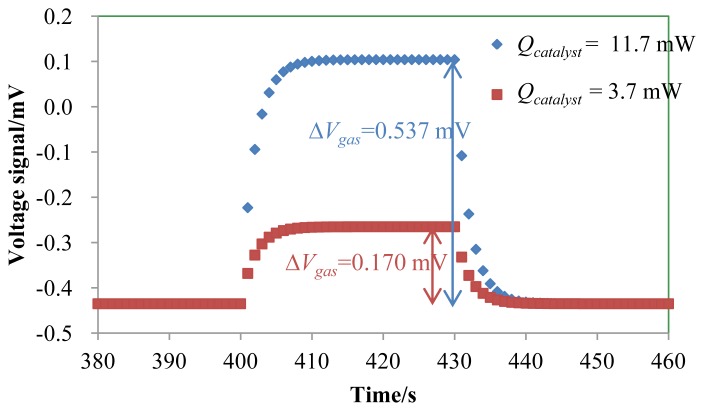
The simulated response curves for Δ*V_gas_* of the TGS for *Q_catalyst_* = 3.7 and 11.7 μW which correspond to 200 and 1,000 ppm H_2_ detection (Figure 4). The other parameters are unchanged and listed in Tables 1 and 2. The simulation results are from 380 to 460 s. *Q_catalyst_* is nonzero from 400 s to 430 s.

Simulations were performed using Equation (15). The flow scheme of the simulation is shown in Figure 5. The simulation time was 600 s, where the heater operation was carried out after 10 s, assuming that the catalyst generates heat from 400 to 430 s. The simulation time step was 1 ms. Figure 6 shows the simulated response curves for *V_S_* of the TGS as a function of *Q_catalyst_* from 5.0 to 15.0 μW. Δ*V_gas_* of the TGS for *Q_catalyst_* = 3.69 and 11.7 μW was 0.230, 0.460, and 0.691 mV, respectively.

The signal curves in Figure 6 matchthe signals in Figure 4. However, Δ*V*_gas_ of the 1,000 ppm hydrogen in Figure 4 increased slightly with increasing time. Because *Q*_catalyst_ increased with increasing catalytic activity since the catalyst temperature increased with the 1,000 ppm H_2_ combustion. In Figure 6, the voltage signal was saturated since *Q*_catalyst_ was constant. In case of the voltage signal of the 200 ppm hydrogen in Figure 2, the catalytic activity was stable since the voltage signal was saturated. It is easy to calculate the voltage signal of TGS with the combustion of the low H_2_.

*R_A_* in this study has been calculated from the time constant of 2 s. Comparing this to the value 0.05 s of our previous report [11], then the new *R_A_* is estimated to be 5.8 K/mW. The reason why the *R_A_* is too large is due to the fact the gas combustion process requires a thermally induced slow reaction and this makes the time constantmuch longer, which leads to a huge apparent thermal resistance. It is originated from the DELAYED time constant problem, which is very unique and a difficult problem of the chemical reaction of catalytic combustion.

**Figure 7.**
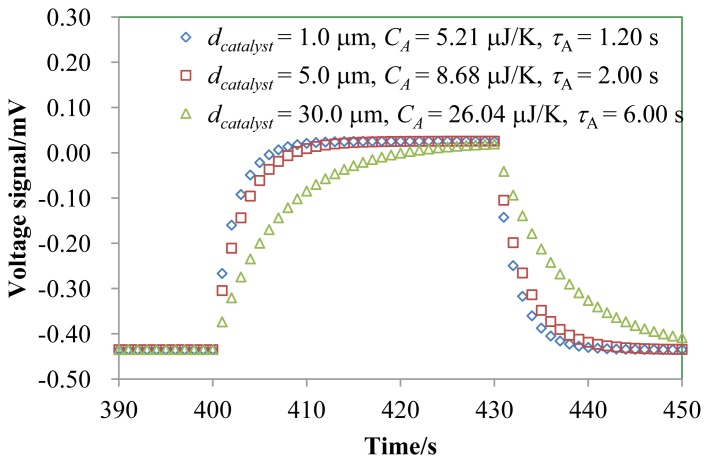
Simulated response curve of the TGS as a function of the catalyst thickness *d_catalyst_*. The catalytic combustion heat *Q_catalyst_* was 10.0 μW.

One important parameter of the gas sensor device is how fast the sensor responds to the gas, *i.e.*, the time constant. We have investigated the effect of the heat capacity of the catalyst thickness *d_catalyst_* on the thermal time constant, as shown in Figure 7.

In our TGS device, a performance enhancement can be achieved by reducing *d_catalyst_*. The increase in the time constant with an increase in the dimension of the catalyst has also been plotted as a function of *d_catalyst_* from 1.00 to 30.0 μm. The values of *τ_A_* for *d_catalyst_* = 1.00, 5.00, and 30.0 μm were 1.2, 2.0 and 6.0 s, respectively. *Q_catalyst_* required for 1.0 mV of Δ*V_gas_* was calculated to be 0.0217 mW. *τ*_H_ is 2 s and *τ*_A_ is 0.1 s. *τ*_H_ is bigger than *τ*_A_ since the supply rate of H_2_ limits the rate of the combustion for H_2_.

## 5. Conclusions

We have analyzed the thermal balance of a micro-TGS, calculated the heat balance at the two sections across a TE film of the TGS device, and estimated the sensor output voltage. *R_A_* and *R_B_* at the two sections were estimated to be 230.4 K/mW and 230.6 K/mW from the thermal time constants of the experimental signal curves. *Q_catalyst_* required for 1 mV of Δ*V_gas_* was calculated to be 0.0217 mW. On the basis of these parameters, simulations for the device performance were performed, and the expected *Q_catalyst_* for 200 and 1,000 ppm H_2_ was 3.69μW and 11.7 μW, respectively.

## Figures and Tables

**Table 1.** Material constants and boundary conditions for the TGS used in this study.

**Parameter**	**Symbol**	**Value**	**Unit**	**References**
Specific heat capacity Pt	-	134	J/(kg·K)	[6]
Density Pt	-	21.45	g/cm^3^	[7]
Specific heat capacity Al_2_O_3_	-	800	J/(kg·K)	“Network database system for thermophysical property data” by AIST and 790 J/(kg·K) is rounded to 800 J/(kg·K).
Density Al_2_O_3_	-	4	g/cm^3^	Product data sheet by Taimei Chemicals Co., Ltd. and 3.95 g/cm^3^ is rounded to 4.00 g/cm^3^.
Dispensed catalytic diameter	-	600	μm	Rounded from the values (565 and 4.64 μm) measured by a KLA-Tencor P16+ Profiler.
Dispensed catalytic thickness	*d_catalyst_*	5	μm	
Dispensed catalytic volume	-	1.41 × 10^−3^	mm^3^	
Seebeck coefficient	*α*	0.2	mV/K	Rounded from the measured value of 0.167 mV/K [3].
Heat capacity catalyst 40wt%Pt/Al_2_O_3_	*C_catalyst_*	4.34	μJ/K	Estimated from the specific heat capacity and density for the catalyst.
Heat capacity for the membrane	*C_membrane_*	4.34	μJ/K	The heat capacity of the membrane in sections A and B was assumed to be the same as the heat capacity of the catalyst.
Heat capacity for section A	*C_A_*	8.68	μJ/K	=*C_catalyst_* +*C_membrane_*
Heat capacity for section B	*C_B_*	4.34	μJ/K	=*C_membrane_*
Ambient temperature	*T_ambient_*	298	K	

**Table 2.** Estimated parameters from the experimental response curve of the TGS for 200 ppm and 1,000 ppm hydrogen (Figure 4). The catalyst of the TGS device was heated up to 120 °C by its micro-heater.

**Parameter**	**Symbol**	**Value**	**Unit**	**Reference**
Voltage signal of the TGS in air	*V_air_*	−0.435	mV	Measured, Assuming that the thickness of Pt heater pattern on the TGS is constant, *Q_heaterA_* and *Q_heaterB_* were designed to be the same and were estimated to be 10.0 mW from the dimensions of the Pt heater pattern measured by the optical microscope image and Joule's laws. As shown in Figure 4, the thermal time constants of two signal curves were the same and equal to 8 s. The gas flow was introduced at steady state after the micro-heater had stabilized. We can then regard the time constant of 2.0 s as *τ_A_*, as described in Equation (15). From *C_A_* in Table 1 and the thermal time constant of section A, *τ_A_* = *R_A_* × *C_A_*, *R_A_* was estimated to be 230.4 K/mW. Using this value for *τ_A_* and *V_air_* = −0.435 mV in Figure 4, *R_B_* was estimated as 230.6 K/mW, and *τ_B_* was calculated as 1 s. *Q_catalyst_* can be calculated from Equation (16) as follows: $Q_{\text{catalyst}}=\frac{\Delta V_{\text{gas}}}{\alpha \cdot R_{A}}=0.0217[W/V]\times \Delta V_{\text{gas}}$
Whole heater power 50 mW	-	50.0	mW	Measured and rounded
Heater power of sections A and B	*Q_heaterA_ Q_heaterB_*	10.0	mW	Estimated from each heater pattern size dimension and the whole heater power 50 mW
Time constant of section A	*τ_A_*	2.0	s	Estimated from gas response of the TGS, Figure 4
Time constant of section B	*τ_B_*	1.0	s	From *τ*_B_ = *R*_B_ × *C*_B_, Equation (14)
Thermal resistance of section A	*R_A_*	230.4	K/mW	From *τ*_A_ = *R*_A_ × *C*_A_, Equation (13)
Thermal resistance of section B	*R_B_*	230.6	K/mW	From *V*_air_ = *α* × *Q*_heater_ × (*R*_A_ – *R*_B_), Equation (9)

* The elapsed time at 63.2% of the saturated Δ*V_gas_*.

## Nomenclature

*P*: heating power input
*T*: temperature
*t*: time
*R*: thermal resistance
*Q_catalyst_*: heat generation of catalytic combustion
*Q_heaterA_*: heat generation of heater for section A
*Q_heaterB_*: heat generation of heater for section B
*T_ambient_*: ambient temperature, mainly room temperature
Δ*T_AB_*: temperature difference between sections A and B
*T_A_*: temperature in section A
*T_B_*: temperature in section B
*C_A_*: heat capacity of section A
*C_B_*: heat capacity of section B
*R_A_*: thermal resistance of section A
*R_B_*: thermal resistance of section B
*V_S_*: voltage signal of the TGS
*V_gas_*: voltage signal in the combustion gas
*V_air_*: voltage signal in air
Δ*V_gas_*: voltage difference between the responses in air and the inflammable gas
*τ_A_*: thermal time constant of section A
*τ_B_*: thermal time constant of section B
